# Ocular Manifestations, Visual Field Pattern, and Visual Field Test Performance in Traumatic Brain Injury and Stroke

**DOI:** 10.1155/2022/1703806

**Published:** 2022-01-07

**Authors:** Yun Jeong Lee, Seung Chan Lee, Seo Young Wy, Hoo Young Lee, Hyang Lim Lee, Woo Hyung Lee, Byung-Mo Oh, Jin Wook Jeoung

**Affiliations:** ^1^Department of Ophthalmology, Seoul National University Hospital, Seoul National University College of Medicine, Seoul, Republic of Korea; ^2^National Traffic Injury Rehabilitation Hospital, Yangpyeong, Republic of Korea; ^3^Department of Rehabilitation Medicine, Seoul National University Hospital, Seoul National University College of Medicine, Seoul, Republic of Korea

## Abstract

**Purpose:**

To analyze ocular manifestations, visual field (VF) pattern, and VF test performance in traumatic brain injury (TBI) and stroke patients.

**Methods:**

This retrospective, cross-sectional study included 118 patients (236 eyes) with TBI and stroke who had undergone VF testing by standard automated perimetry with the central 24-2 threshold test. Clinical features including best-corrected visual acuity (BCVA), intraocular pressure (IOP), ocular manifestations, and VF test results including VF defect pattern, reliability, and global indices were analyzed and compared between the TBI and stroke patients.

**Results:**

In TBI patients, ocular manifestations included strabismus (11.1%), cataract (4.2%), and glaucoma suspect (2.8%), whereas in stroke patients, cataract (15.2%), strabismus (8.5%), diabetic retinopathy (4.9%), extraocular movement (EOM) limitation (3.0%), glaucoma suspect (3.0%), nystagmus (2.4%), drusen (1.2%), and vitreous hemorrhage (1.2%) were found. The VF test results showed that 47 eyes (85.5%) in TBI and 86 (65.2%) in stroke had VF defect; in TBI, the scattered pattern was the most common (56.4%), followed by homonymous hemianopsia (14.5%), homonymous quadrantanopia (10.9%), and total defect (3.6%), whereas in stroke, homonymous hemianopsia was the most common (31.8%), followed by scattered pattern (16.7%), homonymous quadrantanopia (12.1%), and total defect (4.5%). Only 15 eyes (27.3%) in TBI and 32 (24.2%) in stroke showed reliable VF indices. The mean deviation (MD) was −10.5 ± 7.1 dB in TBI and −9.5 ± 6.8 dB in stroke, and the pattern standard deviation (PSD) was 4.9 ± 3.3 dB in TBI and 6.1 ± 3.9 dB in stroke, without statistically significant differences between the two groups.

**Conclusion:**

Various ocular manifestations were found, and a considerable proportion of patients were experiencing VF defects and showed unreliable VF test performance. Our findings suggest that accurate evaluation and rehabilitation of visual function should be a matter of greater concern and emphasis in the management of TBI and stroke patients, besides systemic diseases.

## 1. Introduction

Traumatic brain injury (TBI) and stroke are major causes of death and disability around the world [1, 2]; about 5.3 million people suffer from TBI-related disability in the United States [3], and another 5 million annually are permanently disabled from stroke [4]. Correspondingly, these diseases incur significant socioeconomic and global health burdens, approximately $US400 billion annually for TBI [5] or €27 billion annually in the European Union [6] and $US65.5 billion in the United States in 2008 for stroke [7]. As populations age due to declining mortality resulting from improved healthcare, the number of individuals living with disability, along with the economic burden, is increasing in terms of both TBI and stroke [8–12]. Concomitantly, accurate functional evaluation including visual function, health-related quality of life, and rehabilitation of long-term disabled patients is of growing importance.

TBI- and stroke-related visual dysfunction, which includes sensory dysfunction such as visual acuity and visual field (VF) impairment, motor dysfunction such as strabismus and ocular movement deficit, and perceptual dysfunction, has been reported in several studies [13–23]. In regard to VF assessment of patients, automated perimetry is known to have advantages over manual kinetic perimetry, in that it provides standardized, reproducible results without the need for skilled perimetrists [24, 25] and also presents reliability indices for assessment of the reliability of test results [26].

Although former studies have reported visual dysfunction in cases of TBI and stroke, comparisons of ocular manifestations between TBI and stroke are lacking. Furthermore, only a few studies have analyzed the VF pattern and VF test performance in TBI and stroke or compared them between these two groups. Moreover, most of the VF evaluation studies were based on the confrontation VF test or Goldmann manual kinetic perimetry (GVF) rather than automated perimetry [27]. Therefore, in the present research, we analyzed and compared ocular manifestations, VF pattern, and VF test performance in cases of TBI and stroke.

## 2. Materials and Methods

This retrospective, cross-sectional study was approved by the Institutional Review Board of National Traffic Injury Rehabilitation Hospital and Seoul National University Hospital in South Korea (IRB no. NTRH-20007 and no. 2105-035-1217, respectively), and the study protocol followed the tenets of the Declaration of Helsinki. Informed consent was waived due to the retrospective nature of the study.

In our study, a total of 118 patients (236 eyes) with TBI and stroke who had visited the National Traffic Injury Rehabilitation Hospital and undergone VF testing between 2018 and 2019 were included.

All of the participants underwent a comprehensive ocular examination including best-corrected visual acuity (BCVA), slit-lamp biomicroscopy, Goldmann applanation tonometry, dilated fundus examination, color fundus photography, and standard automated perimetry with the central 24-2 threshold test. Analyses of demographics (age, sex, and underlying disease), clinical features (BCVA, intraocular pressure (IOP), and ocular manifestations), and VF test results (VF defect pattern, VF test reliability index, and VF global index) were conducted as well.

### 2.1. VF Assessment

Among the 118 patients (236 eyes) initially included, those with BCVA of less than 20/40, who have media opacities such as severe cataract or vitreous opacity potentially affecting the VF test results, retinal disease, or glaucoma that could cause VF defect, were excluded from VF assessment. Accordingly, a total of 98 patients (187 eyes) (28 TBI patients, 55 eyes; 70 stroke patients, 132 eyes) were reenrolled. A total of 49 eyes were excluded: 44 due to BCVA of less than 20/40, three due to severe cataract, and two due to vitreous hemorrhage.

During the VF testing, VF test reliability indices (i.e., fixation loss (FL) rate, false positive (FP) rate, false negative (FN) rate, and test duration and VF global indices (i.e., mean deviation (MD) and pattern standard deviation (PSD)) were evaluated. VF results were considered unreliable if FL rates exceeded 20%, FP rates exceeded 15%, or FN rates exceeded 33%. MD and PSD were analyzed in patients with reliable VF results, including 12 TBI patients (15 eyes) and 30 stroke patients (32 eyes).

### 2.2. Statistical Analyses

Descriptive statistics were derived to represent the data, and Student's *t*-test, the chi-square test, Fisher's exact test, and the Mann–Whitney *U* test were used to compare the results between the groups. The data were analyzed using the Statistical Package for the Social Sciences version 23.0 software program (IBM Corp., Armonk, NY, USA). *P* values < 0.05 were considered statistically significant.

## 3. Results

### 3.1. Demographics and Clinical Characteristics

Among the patients included, 36 (30.5%) had been diagnosed with TBI and 82 (69.5%) with stroke. The etiology and types of TBI are shown in Table 1. An analysis of the etiology of TBI revealed that traffic accident was the most common (24 patients, 66.7%), followed by fall down (8, 22.2%), contusion (1, 2.8%), and unknown etiology (3, 8.3%).

Table 2 shows the demographics and clinical features of the TBI and stroke patients with their intergroup comparison. The total mean age was 53.2 ± 17.9 (range, 16.0–89.0 years), and the TBI patients were significantly younger than those with stroke (46.1 vs. 56.2 years, *P* = 0.015). Male patients were more common than females overall (86 patients, 72.9%) and also in both groups, which numbers were 30 (83.3%) in TBI and 56 (68.3%) in stroke (*P* = 0.091).

Ocular manifestations included strabismus, which was more common in stroke than in TBI (7 patients (8.5%) vs. 4 (11.1%), *P* = 0.734). Other features, which were nystagmus and extraocular movement (EOM) limitation, were found only in the stroke group (4 eyes (2.4%) with nystagmus, 5 (3.0%) with EOM limitation); none were noted in the TBI group (*P* = 0.316 for nystagmus, *P* = 0.327 for EOM limitation). Cataract was more common in stroke than in TBI, with statistical significance (25 eyes (15.2%) vs. 3 (4.2%), *P* = 0.015) as were all of the fundus abnormalities, but without statistical significance; as for glaucoma suspect, there were five eyes (3.0%) in the stroke group vs. two (2.8%) in TBI (*P* = 1.000); and as for diabetic retinopathy, drusen, and vitreous hemorrhage, there were eight (4.9%), two (1.2%), and two (1.2%) in the stroke group, respectively, vs. none in the TBI group (*P* = 0.110, *P* = 1.000, and *P* = 1.000, respectively).

### 3.2. VF Testing

Table 3 summarizes the VF test results on the TBI and stroke patients and their intergroup comparison. Among the 28 TBI patients (55 eyes) whose VF test results were analyzed, 47 eyes (85.5%) showed VF defect, in which the scattered pattern was the most common (31 eyes, 56.4%), followed by homonymous hemianopsia (8 eyes, 14.5%), homonymous quadrantanopia (6 eyes, 10.9%), and total defect (2 eyes, 3.6%). By contrast, analysis of the VF defect patterns in the 70 stroke patients (132 eyes) revealed that 86 eyes (65.2%) showed VF defect, in which homonymous hemianopsia (42 eyes, 31.8%) was the most common, followed by the scattered pattern (22 eyes, 16.7%), homonymous quadrantanopia (16 eyes, 12.1%), and total defect (6 eyes, 4.5%). When comparing the VF defect patterns between the two groups, homonymous hemianopsia and normal VF were significantly more common in stroke (*P* = 0.015 and *P* = 0.005, respectively). Homonymous quadrantanopia and total defect were also found to be more common in stroke, though without statistical significance (*P* = 0.815 and *P* = 1.000, respectively). On the other hand, the scattered pattern was significantly more common in TBI than in stroke (*P* < 0.001).

With regard to the VF test reliability indices, TBI showed higher FL and FN rates and longer test duration than stroke, though without statistical significance: FL rate, 43.3 ± 34.0% (range, 0.0–100.0%) in TBI vs. 40.6 ± 29.7% (range, 0.0–100.0%) in stroke (*P* = 0.584); FN rate, 12.8 ± 21.5% (range, 0.0–100.0%) in TBI vs. 11.2 ± 19.0% (range, 0.0–100.0%) in stroke (*P* = 0.622); and test duration, 284.5 ± 74.1 seconds (range, 191.0–538.0 seconds) in TBI vs. 265.3 ± 54.8 seconds (range, 174.0–492.0 seconds) in stroke (*P* = 0.086). However, the FP rate was higher in stroke than in TBI but without statistical significance (6.9 ± 14.8% (range 0.0–88.9%) vs. 4.6 ± 11.9% (range 0.0–66.7%), *P* = 0.302).

Only patients with reliable VF results were selected for VF global index analysis: 12 TBI patients (15 eyes, 27.3%) and 30 stroke patients (32 eyes, 24.2%). The MD value was worse in TBI than in stroke but without statistical significance (−10.5 ± 7.1 dB (range, −29.8 to −1.4 dB) in TBI vs. −9.5 ± 6.8 dB (range, −28.2 to −1.1 dB) in stroke, *P* = 0.508), whereas the PSD value was worse in stroke than in TBI, but again, without statistical significance (6.1 ± 3.9 dB (range, 1.5–12.8 dB) in stroke vs. 4.9 ± 3.3 dB (range, 1.6–12.8 dB) in TBI, *P* = 0.515).

To evaluate the VF test performance in the TBI and stroke patients, the proportion with unreliable VF test results, as determined according to the VF test reliability index, was calculated in each group (Table 4). FL rates of more than 20% during VF testing were found in 34 TBI eyes (61.8%) vs. 88 stroke eyes (66.7%), but without statistical significance (*P* = 0.526). Meanwhile, FP rates of more than 15% were noted in seven TBI eyes (12.7%) vs. 24 stroke eyes (18.2%) (*P* = 0.361), and FN rates of more than 33% were found in eight TBI eyes (14.5%) vs. 15 stroke eyes (11.4%) (*P* = 0.546). Among the three VF test reliability indices, “FL rate of more than 20%” showed the highest proportion in both the TBI and stroke groups.

## 4. Discussion

The ocular manifestations of TBI and stroke in our Korean cohort are in line with previous reports of various structural and functional abnormalities [13–23]. However, the dominant features of each of our groups are somewhat different from the report of Rutner et al. [28], which indicated elevated relative risk ratios of abnormalities unique to each group including corneal abrasion, blepharitis, chalazion, hordeolum, dry eye, traumatic cataract, vitreous prolapse, and optic atrophy in the TBI group and subconjunctival hemorrhage and ptosis in the cerebral vascular accident group. The difference in the types of ocular manifestations between that report and our present study might be attributed to the differences in the examination time from disease onset and in the type, location, and range of damage. With regard, for instance, to the type of damage, the etiology of TBI in the former, US reports dealing with visual dysfunction after TBI commonly includes blast injury [27, 30], whereas in our present cohort, nonblast injury (e.g., traffic accident, fall down, and contusion) accounted for most cases. It should also be noted that although Rutner et al. [28] determined the frequencies and relative risks of ocular diseases in TBI and stroke, they made no statistical comparison between their two groups. Comparing the ocular manifestations between the TBI and stroke groups in our study, there was a higher prevalence of cataract in the stroke group than in the TBI group, which might be attributable to the stroke group's older mean age and a higher rate of systemic diseases including diabetes mellitus and hypertension, as indeed, increasing age and systemic conditions are known risk factors for the development of cataract [31, 32]. Also, the higher prevalence of drusen in the stroke group, though not statistically significant, might have arisen from its older mean age, as in fact, the prevalence of drusen is known to increase with age [33]. Furthermore, since diabetic retinopathy is one of the common complications of diabetes mellitus, its higher prevalence in the stroke group, though not statistically significant either, might have resulted from that the group's higher rate of diabetes mellitus.

With respect to the present VF defect patterns in TBI, the scattered pattern was the most common (56.4%), which is consistent with previous reports by Suchoff et al. (22.5%) [29] and Walsh et al. (48.2%) [30]. Homonymous VF defect was the second most common (25.5%), which is in line with Suchoff et al. (8.75%) [29]; however, Walsh et al. [30] reported that constricted VF defect was the second most common VF defect (6.0%), followed by altitudinal defect (3.0%), hemianopsia (2.4%), and quadrantanopia (1.2%) or central defect (1.2%). Among our cohort of stroke patients, homonymous VF defect was the most common (43.9%), followed by the scattered pattern (16.7%), which findings are consistent with those of Suchoff et al. [29] for cerebral vascular accident patients (homonymous VF defect, 31.67%, followed by scattered defect, 13.33%). Although the main types of VF defect pattern among our TBI and stroke cohorts are similar to those in previous reports, the proportions and types (other than the main pattern) of VF defect differ. These disparities might arise from the differences in the numbers of patients, disease etiology, and/or VF test methods. Moreover, in comparison with the former reports, we included only patients without other abnormalities possibly causing VF defect, in order to evaluate the pure VF defect due to TBI or stroke.

Analysis of our VF test reliability indices indicated that only 27.3% of the TBI and 24.2% of the stroke eyes had reliable VF indices, with FL rates of not more than 20%, FP rates of not more than 15%, and FN rates of not more than 33%. Our results are in comparison to those of Lemke et al.'s study [27], which, to our knowledge, is the only one to have analyzed VF test reliability indices in TBI patients. They concluded that reliable automated perimetry could be accomplished for most of their blast injury-related TBI patients, with only 29% of eyes having shown FLs of more than 20%, none showing FPs of more than 15%, and only 6% showing FNs of more than 33%, with a Humphrey Field Analyzer using the 30-2 SITA-standard or SITA-fast. Also, another study by Szatmáry et al. [34] found that the SITA-fast strategy of automated perimetry was reliable in 77% of eyes of neuro-ophthalmologically impaired patients, which was equal to that of GVF. However, the VF test results had been determined to be unreliable only on the basis of FL rates of 50% or more, rather than including other parameters such as FP or FN rates. Also, since both Lemke et al. [27] and Szatmáry et al.'s [34] studies employed the SITA-fast strategy which could improve cooperation that could have affected the VF reliability results, care should be taken in any attempts to generalize their results to all automated perimetry.

Visual dysfunction is common but often unrecognized or underestimated in cases of TBI and stroke, since patients' care is commonly focused mainly on diagnosis and management of life-threatening systemic diseases. However, since visual deficits could have a great impact on patients' daily living and are known also to have adverse effects on rehabilitation and quality of life [29], accurate evaluation and rehabilitation of visual function are of great importance, besides physical problems.

The strength of our study is that it is one of the few to have systematically analyzed ocular manifestations, VF pattern, and VF test performance in TBI and stroke and to have compared them between two groups within a large study population. Moreover, to our knowledge, this is the first study to have analyzed the ocular features of TBI and stroke in a Korean population. The limitations of our study are its data collection via retrospective analysis of medical records and the lack of follow-up data for analysis of changes of ocular features and VF test results. Further studies with follow-up periods would provide more insights into the natural history of visual dysfunction in TBI and stroke patients. Also, studies comparing VF test performances among different VF test protocols in order to determine the most effective method to improve performance in patients with TBI and stroke would be helpful to clinical practice. Moreover, research on measuring the quality of life of patients as well as their economic burden and visual rehabilitation would provide information invaluable to their care.

## 5. Conclusions

Various ocular manifestations were found, and a considerable proportion of patients were experiencing VF defects and showed unreliable VF test performance. These findings suggest that accurate evaluation and rehabilitation of visual function should be a matter of greater concern and emphasis in the management of TBI and stroke patients, besides systemic diseases.

## Figures and Tables

**Table 1:** Etiology and types of traumatic brain injury.

Variable	No. (%)
Etiology	
Traffic accident	24 (66.7)
Fall down	8 (22.2)
Contusion	1 (2.8)
Unknown	3 (8.3)
Type	
Subdural hemorrhage	21 (34.4)
Subarachnoid hemorrhage	16 (26.2)
Epidural hemorrhage	9 (14.8)
Intracerebral hemorrhage	8 (13.1)
Diffuse axonal injury	5 (8.2)
Intraventricular hemorrhage	2 (3.3)

**Table 2:** Demographics and clinical features in traumatic brain injury and stroke patients.

Variable	Total	Traumatic brain injury (*n* = 72)	Stroke (*n* = 164)	*p* value
Age, yr	53.2 ± 17.9 (16.0–89.0)	46.1 ± 21.8 (16.0–86.0)	56.2 ± 14.9 (22.0–89.0)	**0.015** ^**a**^
Sex (M : F)	86 : 32	30 : 6	56 : 26	0.091^b^
Underlying disease, no. (%)				
Diabetes mellitus^*∗*^		1 (2.8)	18 (22.0)	**0.009** ^**b**^
Hypertension^*∗*^		10 (27.8)	56 (68.3)	**<0.001** ^**b**^
Best-corrected visual acuity (logMAR)		0.18 ± 0.45 (0.0–2.9)	0.036 ± 0.36 (−1.0–2.9)	**0.015** ^**a**^
Intraocular pressure (mmHg)		14.7 ± 3.1 (9.0–23.0)	14.9 ± 3.0 (7.0–25.0)	0.728^a^
Ocular manifestations, no. (%)				
Strabismus^*∗*^		4 (11.1)	7 (8.5)	0.734^c^
Nystagmus		0 (0.0)	4 (2.4)	0.316^c^
Extraocular movement limitation		0 (0.0)	5 (3.0)	0.327^c^
Cataract		3 (4.2)	25 (15.2)	**0.015 ** ^**b**^
Fundus abnormality				
Glaucoma suspect		2 (2.8)	5 (3.0)	1.000^c^
Diabetic retinopathy		0 (0.0)	8 (4.9)	0.110^c^
Drusen		0 (0.0)	2 (1.2)	1.000^c^
Vitreous hemorrhage		0 (0.0)	2 (1.2)	1.000^c^

Data are mean ± standard deviation (range) unless otherwise indicated. ^a^Student's *t*-test. ^b^Chi-square test. ^c^Fisher's exact test. ^*∗*^*n* = 36 for TBI and *n* = 82 for stroke. Bold indicates *P* < 0.05.

**Table 3:** Visual field test results in traumatic brain injury and stroke patients.

Variable	Traumatic brain injury (*n* = 55)	Stroke (*n* = 132)	*p* value
Visual field defect pattern, no. (%)			
Homonymous hemianopsia	8 (14.5)	42 (31.8)	**0.015** ^**a**^
Homonymous quadrantanopia	6 (10.9)	16 (12.1)	0.815^a^
Total defect	2 (3.6)	6 (4.5)	1.000^b^
Scattered	31 (56.4)	22 (16.7)	**<0.001** ^**a**^
Normal	8 (14.5)	46 (34.8)	**0.005** ^**a**^
Visual field test reliability index			
Fixation loss (%)	43.3 ± 34.0 (0.0–100.0)	40.6 ± 29.7 (0.0–100.0)	0.584^c^
False positive (%)	4.6 ± 11.9 (0.0–66.7)	6.9 ± 14.8 (0.0–88.9)	0.302^c^
False negative (%)	12.8 ± 21.5 (0.0–100.0)	11.2 ± 19.0 (0.0–100.0)	0.622^c^
Test duration (sec)	284.5 ± 74.1 (191.0–538.0)	265.3 ± 54.8 (174.0–492.0)	0.086^c^
Visual field global index			
Mean deviation (dB)^*∗*^	−10.5 ± 7.1 (−29.8–−1.4)	−9.5 ± 6.8 (−28.2–−1.1)	0.508^d^
Pattern standard deviation (dB)^*∗*^	4.9 ± 3.3 (1.6–12.8)	6.1 ± 3.9 (1.5–12.8)	0.515^d^

Data are mean ± standard deviation (range) unless otherwise indicated. ^a^Chi-square test. ^b^Fisher's exact test. ^c^Student's *t*-test. ^d^Mann–Whitney *U* test. ^*∗*^*n* = 15 for TBI and *n* = 32 for stroke. Bold indicates *P* < 0.05.

**Table 4:** Visual field test reliability index in traumatic brain injury and stroke patients.

Variable	Traumatic brain injury (*n* = 55)	Stroke (*n* = 132)	*p* value
Fixation loss (%) > 20	34 (61.8)	88 (66.7)	0.526^a^
False positive (%) > 15	7 (12.7)	24 (18.2)	0.361^a^
False negative (%) > 33	8 (14.5)	15 (11.4)	0.546^a^

^a^Chi-square test.

## Data Availability

The data used to support the findings of this study are available from the corresponding author upon request.
